# Torsion of the Retroperitoneal Kidney: Uncommon or Underreported?

**DOI:** 10.1155/2014/561506

**Published:** 2014-01-16

**Authors:** Michael Sosin, Wuya Lumeh, Matthew Cooper

**Affiliations:** ^1^Medstar Georgetown Transplant Institute, Georgetown University Hospital, 3800 Reservoir Road, NW, Main Hospital Building, Second Floor, Washington, DC 20007, USA; ^2^Georgetown University School of Medicine, Medical Dental Building, 3900 Reservoir Road, NW, Washington, DC 20057, USA

## Abstract

Vascular torsion in a renal allograft after placement in the retroperitoneum is rare and has only been reported twice in the literature. It is an extrinsically mediated process that occurs at the vascular pedicle resulting in graft compromise and potential loss. Rapid diagnosis and immediate surgical intervention may salvage allograft function. Herein, we present a unique case of a 42-year-old male that developed renal allograft torsion following a second kidney transplant placed in the retroperitoneum. Immediate detorsion did not resolve allograft dysfunction, and a biopsy revealed acute cellular mediated rejection. After antithymocyte globulin treatment, allograft function was salvaged. A review of the current literature shows that the incidence, morbidity, and long term allograft function of intraperitoneal and extraperitoneal torsion are different. As such, torsion of the retroperitoneal kidney demonstrates encouraging allograft salvage rates. Only the third case reported to date, this serves as a contribution to the growing body of literature in retroperitoneal renal torsion and reviews the risks, medication considerations, diagnostic tests, and treatment modalities in a unique disease process.

## 1. Introduction

Commonly reported short-term complications following kidney transplant include postoperative hemorrhage, thrombosis, urine leak, ureteral stricture, and acute rejection [1, 2]. Thrombosis can occur from intrinsic mediators or extrinsic factors that compromise vascular inflow or outflow [3]. Vascular torsion is an extrinsically mediated, rare, and potentially reversible complication that occurs at the vascular pedicle resulting in graft compromise and/or loss. Rapid diagnosis and immediate surgical intervention may salvage allograft function. However, a delay in diagnosis increases the risk of acute rejection or frank necrosis sometimes requiring transplant nephrectomy [4, 5]. Herein, we present a case complicated by renal torsion and subsequent acute rejection. Early recognition and treatment of both complications allowed for successful salvage of the graft. This case serves as only the third report of renal hilar torsion in retroperitoneal placement of a kidney allograft [6, 7].

## 2. Case Report

A 42-year-old male with hemodialysis-dependent (HD), end stage renal disease (ESRD) underwent a second deceased donor kidney transplant (DDKT). His past medical history included glomerulonephritis, the etiology of his ESRD, for which he underwent an initial DDKT at the age of 30. Following this first transplant, the patient was diagnosed with donor-derived metastatic melanoma. Immediate discontinuation of immunosuppression was followed by a donor nephrectomy that same year. The patient was disease-free without evidence of metastasis for the following 11 years. In lieu of a prior malignancy, he did not undergo center-standard induction therapy with alemtuzumab and instead received 500 mg of intravenous methylprednisolone and 40 mg of intravenous basiliximab. The standard criteria donor was a 20-year-old male that suffered from head trauma following a motor vehicle accident. Cold ischemia time was 4 hours. Incision and placement of the allograft was in the left retroperitoneal iliac fossa due to the patient's prior right sided surgeries. The donor had an accessory renal artery and a single arterial anastomosis was completed using a single aortic cuff receiving inflow from both tributary vessels. The venous anastomosis was completed in standard fashion. Operative time was 1.5 hours. Intraoperative urine output was 225 mL and postoperatively continued at a rate of 40–500 mL/hr for the following 10 hours. Urine output then began to decrease acutely from 25 mL/hr to 10 mL/hr. Renal ultrasound obtained at postoperative hour 11 demonstrated elevated velocity at the main renal artery anastomosis. A loss of tardus parvus waveform with a decreased resistive index was concerning for external compression, kink, stenosis, or impending thrombosis. No perinephric fluid collection was visible. The concern for a technical error prompted immediate return to the operating room on postoperative day (POD) 1. Intraoperatively, the allograft was torsed medially across the vascular pedicle with mild discoloration suggestive of ischemia. No evidence of frank necrosis was observed. After detorsion, intraoperative ultrasound assessment confirmed reestablishment of arterial and venous flow. A biopsy was not performed at the time of reexploration. Nephropexy of the superior pole to adjacent soft tissue and the lateral abdominal wall was then completed. The iliac fossa space was more intimately closed to decrease the potential space.

The patient remained oliguric requiring hemodialysis with ongoing elevations in serum creatinine. With a concern for rejection, on hospital day 6 the patient underwent an ultrasound-guided biopsy with final pathology revealing Banff 2B acute cellular rejection. A 5-day cycle of anti-thymocyte globulin treatment was initiated with the patient producing urine after 18 hours of treatment. The creatinine level markedly decreased from 6 at initiation of treatment to 1.35 upon discharge. Throughout his perioperative and postoperative course, the immunosuppression regimen consisted of prednisone, mycophenolate mofetil beginning on POD 2, and tacrolimus was initiated on POD 8.

## 3. Discussion

Torsion of the transplanted allograft is a rare complication. Only two reports of vascular torsion in a renal allograft have been reported in retroperitoneal transplants [6, 7]. The majority of vascular torsion has been reported in the intra-abdominally placed kidney associated with simultaneous pancreas kidney (SPK) transplants [8]. The purported risk factors for intraperitoneal renal torsion include pediatric transplant recipients, patients with Prune Belly syndrome, and patients taking chronic corticosteroids [9–14]. Immunosuppressive medication is believed to induce an adhesion-free intraabdominal environment, acknowledging a potential role for nephropexy to adjacent structures. Although multiple authors recommend prophylactic nephropexy for SPK transplantation, there is a paucity of data to support it in retroperitoneal kidney transplantation [8, 12, 13]. Modi et al. have reported 2 of 6 patients undergoing a laparoscopic kidney transplant without nephropexy developed renal torsion and subsequently underwent nephrectomy. The following 38 cases completed by the same group utilized laparoscopic prophylactic nephropexy yielding a 0% torsion rate [15]. Despite such promising findings, a randomized control trial would better elucidate the efficacy of such practice in open SPKs or open retroperitoneal kidney transplants.

Torsion has been described to occur as early as POD 1, as evidenced in this case, to as long as 10 years postoperatively [16]. Lucewicz et al. provide a detailed description of varying mechanisms of torsion in a review of 16 intraperitoneal cases. In this case, the mechanism of twist occurred anteromedially, from left to right along the sagittal axis. Rotation along the coronal or transverse axis ranging from 120 to 360 degrees has also been described [9, 10, 12, 13, 16, 17]. We attributed our complication to a large potential space in the iliac fossa. However, other possible explanations include a long vascular pedicle, long ureter, increased laxity in the abdominal wall, or excessive fluid in the perinephric space.

Lymphocele is the most common perinephric fluid collection following extraperitoneal kidney transplants, and it has a lower incidence in intraperitoneally placed transplants because of the absorptive capacity of the peritoneum [18]. Fenestration techniques have been advocated for prevention of retroperitoneal fluid collections [19, 20]. However, Zaontz and Firlit only studied pediatric patients [19]. Syversveen et al. defined lymphocele so broadly that interpretation of other perinephric fluid collections may have been misdiagnosed as lymphoceles [20]. To date there is insufficient evidence to support prophylactic fenestration for prevention of renal torsion.

Renal allograft ultrasonography is a routine method of serially monitoring for vascular complications. Including color flow Doppler imaging is a rapid, inexpensive, and accurate method of assessing vascular patency. The overall sensitivity of this method when evaluating the renal pedicle is 95%, with specificity of 92% [6]. Computerized tomography angiography (CTA) and magnetic resonance imaging angiography (MRA) are superior methods of imaging but are costly and may delay intervention in the acute setting. In the setting of a nonacute, subclinical presentation of kidney torsion, MRA provides a reconstructed 3-dimensional image and can serve as an excellent modality in diagnosing renal torsion as an etiology of renal artery stenosis, hypertension, and chronic rejection [4].

Once recognized, treatment involves immediate detorsion in the operating room. Rates of graft salvage after detorsion are approximately 44%. Immediate nephrectomy has been reported in 38% of torsion cases, with 19% of delayed allograft loss following detorsion [8]. Exclusive to intraabdominal torsion, these rates differ from retroperitoneal torsion case reports. Including our case, now 3 retroperitoneal torsed kidneys have been reported to have a 100% salvage rate in allograft function after detorsion. We do recognize that this may be overly optimistic and is likely subject to publication bias.

Intraoperative findings confirmed torsion as the etiology of our patient's acute onset of oliguria. A biopsy was deferred as the source of allograft dysfunction seemed to be apparent and suspicion of acute rejection was low. In retrospect, a biopsy should have been obtained as the risk of acute cellular mediated rejection increases in the setting of renal ischemia [21]. This episode served as a second warm ischemic episode thereby increasing the risk of rejection. Despite rapid restoration of allograft perfusion, ensuing graft dysfunction prompted a kidney biopsy confirming Banff 2B rejection. Upon treatment with anti-thymocyte globulin, allograft function returned within 18 hours, ultimately salvaging allograft function and avoiding the loss of our patient's second and potentially final transplant.

Exceptional to this case was the patient's prior history of a donor-transmitted malignancy. Attenuated immunosuppressive regimens have been advocated for transplant recipients with prior malignancy [22, 23]. In lieu of his donor-transmitted metastatic melanoma, we avoided aggressive induction therapy. Additionally, multiple trips to the operating room and poor renal function altered our standardized immunosuppressive protocol.

This case underscores the importance of maintaining a high suspicion of renal torsion despite placement of the allograft in the retroperitoneum. Salvage rates after detorsion dramatically differ from that of intraperitoneally placed allografts suggesting that retroperitoneal torsion may result in better outcomes after detorsion and should be studied as a distinct entity. We also emphasize the importance of obtaining a biopsy upon detorsion of the graft. It is unclear whether this was an isolated technical complication as the etiology for rejection, whether an attenuated immunosuppressive regimen was a culpable source, or both issues contributed to the outcome. We describe a series of rare events occurring in one case. Circumventing a complete workup can only be pursued with caution and in retrospect may delay a diagnosis. After detorsion, if transplant nephrectomy is avoided, we recommend obtaining a biopsy in the operating room. We emphasize the fundamentals of a thorough evaluation following persistent graft dysfunction are of paramount importance in salvaging a threatened renal allograft.
